# Supplementary material for: A Commercial Probiotic Induces Tolerogenic and Reduces Pathogenic Responses in Experimental Autoimmune Encephalomyelitis
Source: Cells. 2020 Apr 7;9(4):906. doi: 10.3390/cells9040906 (PMC7226819; doi:10.3390/cells9040906)
Supplement: Supplementary file 1 [file cells-09-00906-s001.zip › FigureS1_proofs.docx]

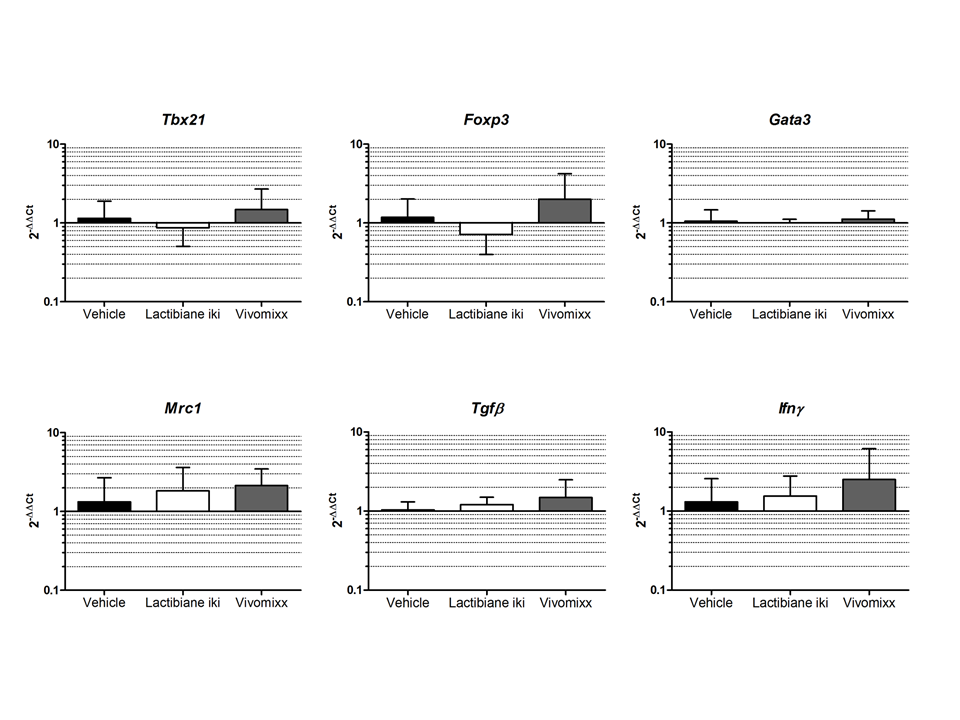


**Figure S1.** Multispecies probiotics do not alter the main transcription factors and cytokines related to experimental autoimmune encephalomyelitis (EAE) pathogenesis in the central nervous system (CNS). Total RNA was extracted from the spinal cords of euthanized EAE mice at 34 days postimmunization (dpi). Next, genomic DNA trace was removed and mRNA was reverse transcribed. Primers for *Tbx21*, *Foxp3*, *Gata3*, *Mrc1*, *Tgfb1*, *Ifnγ*, *Il4*, *Il17a*, *Il10* and the housekeeping gene *Gapdh* were selected as key pathogenic genes of EAE and their relative level of gene expression was calculated using the 2^−ΔΔCT^ method. None of the commercial probiotics, Lactibiane iki and Vivomixx, alter the expression of the transcription factors defining Th1 cells (*Tbx21*), regulatory T (T_reg_) cells (*Foxp3*), Th2 cells (*Gata3*) or type 2 macrophages (*Mrc1*) or the cytokines *Tgfβ* and *Ifnγ* related to the main immune responses in the EAE model. On the other hand, several cytokines were even not detected (*Il17a*, *Il4*, *Il10*) (*data not shown*). The graphs show the results of a representative experiment under double dose administration (vehicle, n = 9; Lactibiane iki, n = 8; and Vivomixx, n = 9). The data are presented as the means ± standard deviations.
