# Supplementary material for: A Commercial Probiotic Induces Tolerogenic and Reduces Pathogenic Responses in Experimental Autoimmune Encephalomyelitis
Source: Cells. 2020 Apr 7;9(4):906. doi: 10.3390/cells9040906 (PMC7226819; doi:10.3390/cells9040906)
Supplement: Supplementary file 1 [file cells-09-00906-s001.zip › FigureS3_proofs.docx]

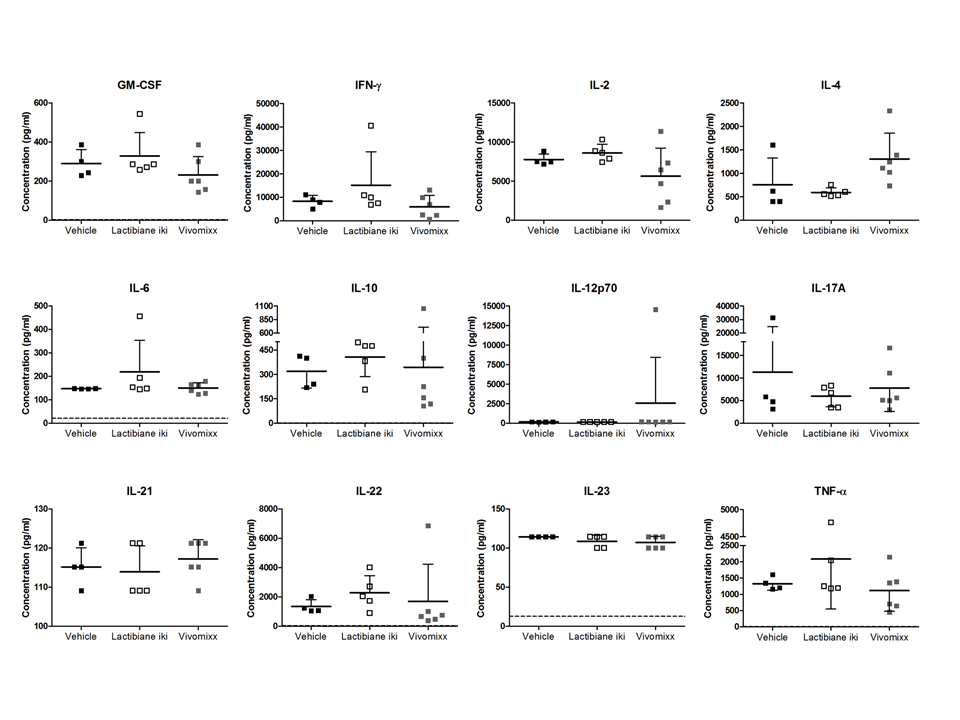


**Figure S3.** Multispecies probiotics do not modify cytokine secretion pattern in the supernatant of proliferating polyclonal cells. Splenocyte suspensions were prepared by grinding spleens of experimental autoimmune encephalomyelitis (EAE) mice through a 70-μm nylon cell strainer at 34 days postimmunization (dpi). Splenocytes were seeded within 96-well plates and stimulated with 5 μg/ml of phytohaemagglutinin-L (PHA-L) and compared to non-stimulated (control) condition. After 54 h *in vitro*, 75 μl of supernatant were harvested and stored at −80 °C to further assess cytokine secretion. Cytokine secretion pattern (granulocyte-macrophage colony-stimulating factor (GM-CSF), interferon gamma (IFN-γ), interleukin (IL)-2, IL-4, IL-6, IL-10, IL-12p70, IL-17A, IL-21, IL-22, IL-23 and tumour necrosis factor alpha (TNF-α)) was assessed in the supernatants of PHA-L-stimulated splenocytes by using a ProcartaPlex Multiplex Immunoassay. Nor Lactibiane iki nor Vivomixx treatment alter cytokine secretion pattern in the supernatant of PHA-L-stimulated splenocytes, as assessed at the end of the experiment (34 dpi). The graphs show the results of a representative experiment under single dose administration (vehicle, n = 4; Lactibiane iki, n = 5; and Vivomixx, n = 6). The dotted lines represent the limit of quantification. The data are presented as the means ± standard deviations. Abbreviations: GM-CSF: granulocyte-macrophage colony-stimulating factor; IFN: interferon; IL: interleukin; TNF-α: tumour necrosis factor alpha.
