# Supplementary material for: A Commercial Probiotic Induces Tolerogenic and Reduces Pathogenic Responses in Experimental Autoimmune Encephalomyelitis
Source: Cells. 2020 Apr 7;9(4):906. doi: 10.3390/cells9040906 (PMC7226819; doi:10.3390/cells9040906)
Supplement: Supplementary file 1 [file cells-09-00906-s001.zip › FigureS4_proofs.docx]

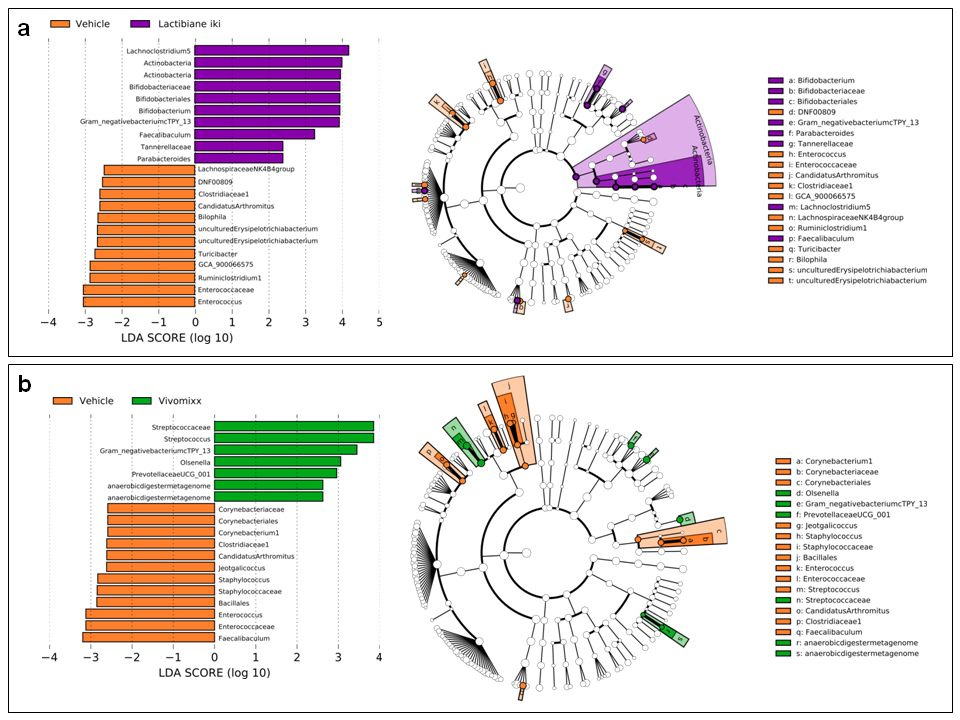


**Figure S4.** Multispecies probiotics alter the taxonomic group abundances in the gut microbiome. Faeces were freshly collected in duplicate from every treated experimental autoimmune encephalomyelitis (EAE) mouse (n=8 per group of treatment: Lactibiane iki, Vivomixx or vehicle) at 33 days postimmunization (dpi). After collection, samples were frozen by immersion in liquid nitrogen and stored at -80ºC. Once 16S rDNA sequencing and quality controls of total faecal samples were performed, the relative abundance of specific taxonomic groups was analysed between experimental groups. Lactibiane iki administration increases the relative abundance of several taxa, including *Lachnoclostridium* and *Bifidobacterium* (**a**), whereas Vivomixx treatment enhances different microbial taxa, such as *Streptococcus* (**b**), compared to vehicle treatment. LEfSe was used to test taxonomic comparisons. In addition to detecting significant features, LEfSe also ranks features by the effect size, assigning higher ranks to features that explain more of the biological difference. The alpha value was set at 0.05, and the threshold for the logarithmic LDA score for discriminative taxa was set at 2.0. The graphs show the results of a representative experiment under double dose administration (vehicle, n = 8; Lactibiane iki, n = 8; and Vivomixx, n = 8).
