# Supplementary material for: A Commercial Probiotic Induces Tolerogenic and Reduces Pathogenic Responses in Experimental Autoimmune Encephalomyelitis
Source: Cells. 2020 Apr 7;9(4):906. doi: 10.3390/cells9040906 (PMC7226819; doi:10.3390/cells9040906)
Supplement: Supplementary file 1 [file cells-09-00906-s001.zip › FigureS5_proofs.docx]

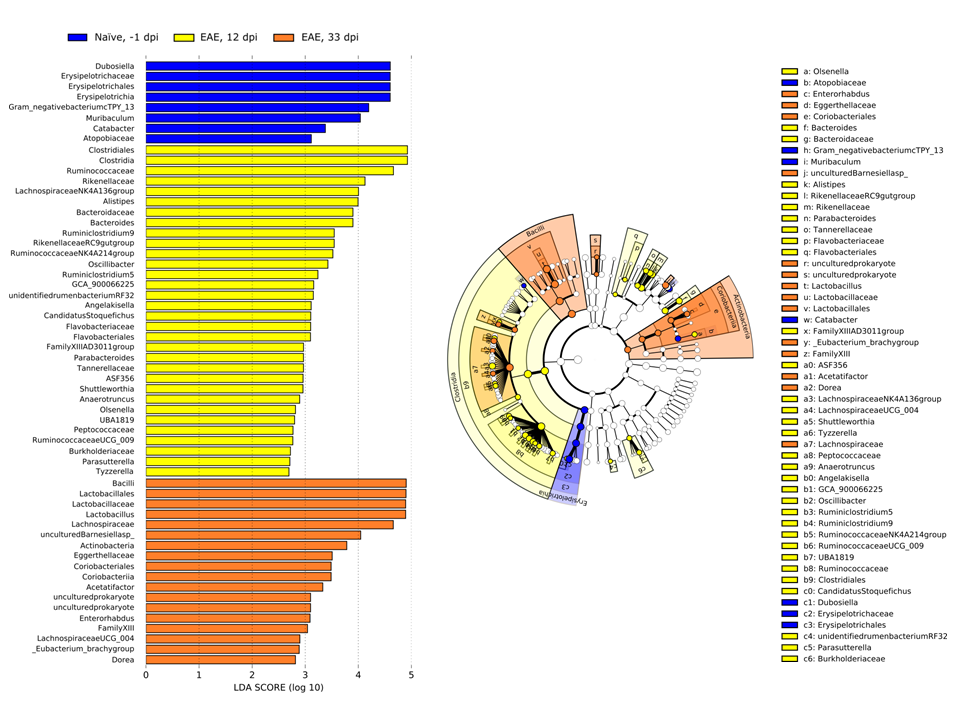


**Figure S5.** The clinical condition modifies taxonomic group abundances in the gut microbiome. Faeces were freshly collected in duplicate from representative mice at -1 days postimmunization (dpi) (n = 12, untreated naïve mice) and 12 dpi (n = 12, untreated experimental autoimmune encephalomyelitis (EAE) mice), considering the cage, clinical score and cumulative score when appropriate. Faecal samples were also freshly gathered in duplicate from every EAE mouse of the vehicle group (n = 8, vehicle-treated mice) at 33 dpi. After collection, samples were frozen by immersion in liquid nitrogen and stored at -80ºC. Once 16S rDNA sequencing and quality controls of total faecal samples were performed, the relative abundance of specific taxonomic groups was analysed between experimental groups. Compared to naïve mice, EAE mice at 12 dpi exhibit an increased abundance of several taxa belonging to the orders *Clostridiales* and *Bacteroidales* (class *Clostridia*), while EAE mice at 33 dpi exhibit an increase in bacteria belonging to the orders *Lactobacillales* (class *Bacilli*) and *Clostridiales* (class *Clostridia*). LEfSe was used to test taxonomic comparisons. In addition to detecting significant features, LEfSe also ranks features by effect size, assigning higher ranks to features that explain more of the biological difference. The alpha value was set at 0.05, and the threshold on the logarithmic LDA score for discriminative taxa was set at 2.0. The graphs show the results of a representative experiment (naïve, -1 dpi, n = 12; EAE, 12 dpi, n = 12; and EAE, 33 dpi, n = 8).
